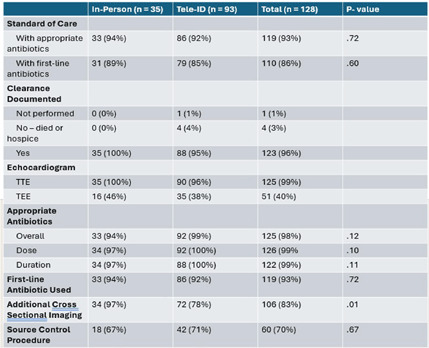# Tele-ID Consultation is Associated with Receipt of Standard of Care and Decreased Mortality in Staphylococcus Aureus Bacteremia

**DOI:** 10.1017/ash.2025.236

**Published:** 2025-09-24

**Authors:** Michael Zou, Sharon Onguti, George Nelson, Nico Herrera, Milner Staub, Titus Daniels, Zhiguo Zhao

**Affiliations:** 1 Vanderbilt University; 2 Vanderbilt University Medical Center; 3 VUMC; 4 University of Chicago Medicine

## Abstract

**Background:** Most people in the US lack access to infectious disease (ID) expertise, with 80% of counties lacking an ID physician. This is problematic as in-person ID consultation has been shown to improve clinical outcomes such as mortality with certain invasive infections, with Staphylococcus aureus bacteremia (SAB) as the paradigm. Telemedicine consultation has emerged as a tool to expand access in rural and underserved communities though its impact on clinical outcomes is less well established. This study characterizes the impact of a Tele-ID program in improving care for patients with SAB at a network of academic-affiliated rural hospitals that do not have access to in-person ID consultation. **Methods:** This was a retrospective cohort study of patients with SAB who were initially evaluated at 3 academic-affiliated rural hospitals between 7/1/22 and 6/30/24. A cohort of patients who received a Tele-ID consult was compared against a cohort that did not. The primary outcome was adherence to the standard of care for SAB, defined as documentation of clearance of blood cultures, receipt of an echocardiogram, and receipt of an appropriate course of antibiotics. Secondary outcomes included clinical outcomes such as mortality and readmission rates. **Results:** A total of 260 discrete episodes of SAB were screened for inclusion, with 122 episodes meeting inclusion criteria. Seventy five patients (61.5%) who received a Tele-ID consult were compared against 47 patients (38.5%) who did not. Patient characteristics were overall similar in these groups, though those receiving Tele-ID consultation were more likely to have end-stage renal disease (15% vs 0%, p < .01) and indwelling hardware (56% vs 21%, p < .01). Tele-ID consultation was associated with a higher likelihood of receiving standard of care for SAB (91% vs 15%, p < .01). This finding was consistent across all hospitals and among the individual components of the primary outcome. In addition, Tele-ID consultation was associated with significantly decreased SAB-related 30-day mortality (7 vs 24%, p < .01) and SAB-related 90-day mortality (8 vs 25%, p < .01). No significant difference was observed in rates of readmission or relapsed bacteremia. **Conclusion:** In this retrospective cohort study of 122 patients with SAB cared for in rural, academic-affiliated hospitals, Tele-ID consultation was associated with a significantly increased likelihood of receiving standard of care and decreased mortality. This data will inform policy at regional hospitals, such as supporting a mandatory ID consult for SAB and implementation of a SAB bundle.

**Figure:**
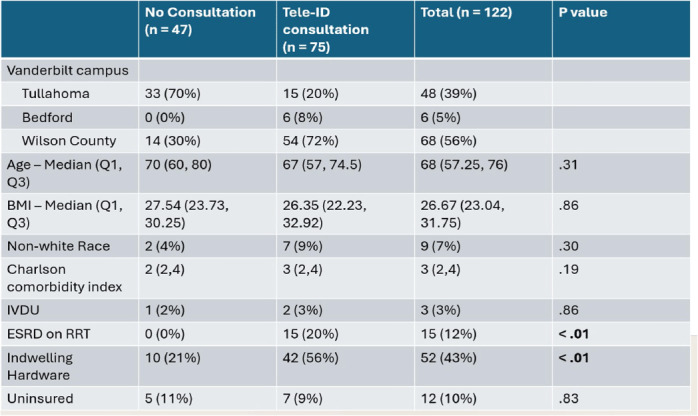

**Figure:**
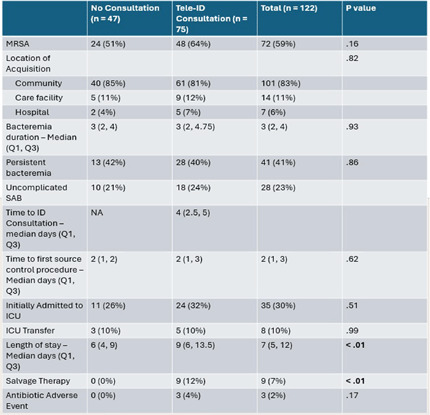

**Figure:**
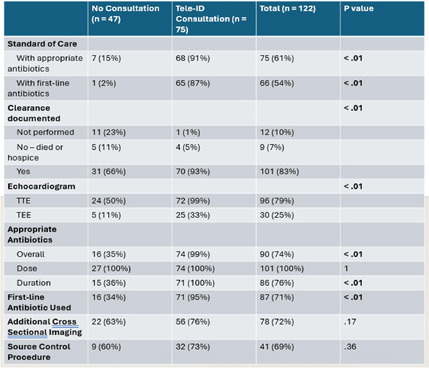

**Figure:**
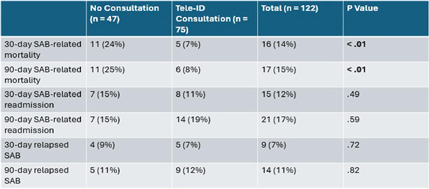

**Figure:**
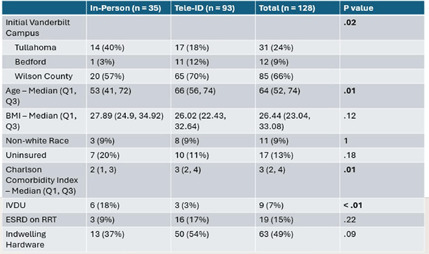

**Figure:**
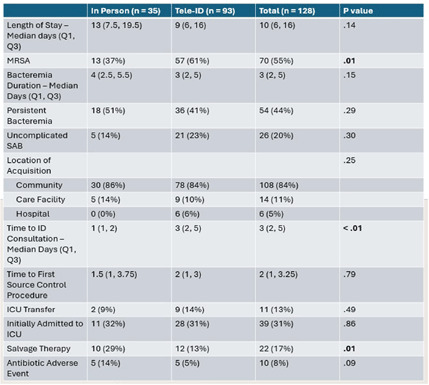

**Figure:**